# Anterior Segment Optical Coherence Tomography Changes to the Anterior Chamber Angle in the Short-term following Laser Peripheral Iridoplasty

**DOI:** 10.5005/jp-journals-10008-1152

**Published:** 2014-01-16

**Authors:** James Chiung Yoong Leong, Jeremy O'Connor, Ghee Soon Ang, Anthony P Wells

**Affiliations:** Registrar, Department of Ophthalmology, Capital and Coast District Health Board, New Zealand; Glaucoma Fellow, Department of Ophthalmology, Royal Victoria Eye and Ear Institute, Australia; Consultant Ophthalmologist, Department of Ophthalmology, Royal Victoria Eye and Ear Institute, Australia; Consultant Ophthalmologist, Department of Ophthalmology, Capital Eye Specialists, New Zealand

**Keywords:** Optical coherence tomography, Angle closure glaucoma, Laser peripheral iridoplasty.

## Abstract

**Purpose:** To evaluate, by anterior segment optical coherence tomography (AS-OCT), the changes in the anterior chamber angle during the short-term postoperative period after diode laser peripheral iridoplasty (LPI).

**Methods:** Retrospective, observational study of consecutive primary angle closure suspect, primary angle closure, or primary angle closure glaucoma patients who underwent LPI. These patients had persistent iridotrabecular contact despite the presence of a patent peripheral iridotomy.

The AS-OCT images of the temporal and nasal anterior chamber angles in dark conditions before and after LPI were ana lyzed. The main outcome measures were changed in AS-OCT parameters such as trabecular-iris angle (TIA), angle opening distance (AOD), trabecular-iris space area (TISA), trabecular-iris contact length (TICL), iris thickness (IT), and maximum iris bow height (MIBH). Secondary outcome para meters included intraocular pressure (IOP) and postlaser complications.

**Results:** Images of 14 eyes of 14 patients were assessed. The mean time from LPI to the follow-up AS-OCT scan was 6 ± 3 weeks. The IT did not alter significantly after LPI, but there were significant increases in the TIA, AOD and TISA, as well as a significant decrease in TICL and MIBH. There were no significant postlaser complications. There was a small decrease in mean IOP from 17.1 ± 4.0 mm Hg to 14.8 ± 4.6 mm Hg (p = 0.014).

**Conclusion:** Based on AS-OCT imaging, LPI resulted in significant angle widening and iris profile fattening during the short-term postoperative period in eyes with persistent angle closure despite the presence of a patent peripheral iridotomy.

**How to cite this article:** Leong JCY, O'Connor J, Ang GS, Wells AP. Anterior Segment Optical Coherence Tomography Changes to the Anterior Chamber Angle in the Short-term following Laser Peripheral Iridoplasty. J Current Glau Prac 2014;8(1):1-6.

## INTRODUCTION

Glaucoma is the leading cause of irreversible blindness worldwide.^1^ Angle closure glaucoma is the main cause of visual morbidity from glaucoma in Asian populations, thought to blind 10 times more people than primary open angle glaucoma.^2^ However, angle closure glaucoma is also a significant, and likely underdiagnosed, condition in Caucasians.^3^

In the setting of primary angle closure (PAC), the recommended initial nonsurgical means of widening the anterior chamber drainage angle is by laser peripheral iridotomy (LPI), which eliminates pupil block.^4^ However, a proportion of eyes will still have residual angle closure, despite a successfully performed and patent PI.^5^ In such patients, LPI may be a useful treatment to ameliorate appo sitional angle closure that may be occurring through nonpupil block mechanisms.^6-8^ It is thought to achieve this by applying a thermal energy which contracts the peripheral iris away from the trabecular meshwork while, also causing thinning of the anterior iris in the treatment spots, thereby helping to further open the anterior chamber angle. While the strength of current evidence with regard to overall efficacy of LPI remains weak,^9^ at present the consensus among glaucoma experts is that it remains a useful adjunctive treatment tool for angle closure.^10^

Anterior segment optical coherence tomography (AS-OCT) is a noncontact imaging modality that rapidly obtains high resolution cross section images of the anterior segment with the patient seated upright. These features are advan tageous in comparison to older imaging modalities used for assessing angle closure, such as ultrasound biomicroscopy (UBM).^11^ Previous work by this group utilized AS-OCT to outline changes in anatomical features of the anterior chamber angle after laser PI in a cohort of patients with angle closure.^12,13^ Changes in angle configuration following PI have been previously well-described using AS-OCT in various other clinical populations.^14-16^

LPI has been shown by gonioscopy to result in widening of the anterior chamber angle in patients with residual angle closure despite a patent laser PI.^17^ However, there has been no direct, quantitative documentation of this angle widening effect with AS-OCT, apart from a few isolated case reports.^18^ With this in mind, the aim of this study was to describe the quantitative changes to the anterior chamber angle after LPI in a cohort of patients with residual appositional angle closure after PI.

## MATERIALS AND METHODS

This was a retrospective case series of patients who underwent diode LPI at Capital Eye Specialists, Wellington, New Zealand over a 21-month period. This cohort of patients was derived from a previously described cohort of 71 eyes of 71 patients who had undergone laser PI for primary angle closure suspect (PACS) status, PAC and primary angle closure suspect (PACG).^13^ Of this original cohort of 71 eyes, 14 (19.7%) underwent subsequent LPI for persistent iridot rabecular contact despite a patent PI, forming the cohort for this study.

Materials and Methods used for the initial cohort are outlined in a previous paper.^12^ Briefy, in the initial cohort, a full ocular examination was performed for each newly referred patient with suspected angle closure. This included best-corrected Snellen acuity, slit-lamp evaluation, Goldmann applanation tonometry, corneal pachymetry, undilated fundoscopy, gonioscopy, and time domain AS-OCT imaging. The AS-OCT was performed by ophthalmic imaging technicians with the slit-lamp OCT (Heidelberg Engineering, GmBH, Dossenheim, Germany) in both uniform light and dark (all room lights switched off) conditions, with scans being centered on the pupil. The clinical decision as to whether the PI was indicated in these patients was made by the glaucoma specialist (APW) according to AS-OCT and gonioscopic findings. The gonioscopic threshold for PI was nonvisibility of the trabecular meshwork in at least 180° of the anterior chamber angle, consistent with the Association of International Glaucoma Societies consensus on angle closure gonioscopic criteria.^10^ The AS-OCT threshold was extrapolated from these criteria as being the presence of iridotrabecular contact, visualized as apposition of peripheral iris to the inner corneoscleral wall anterior to the scleral spur, in at least two of four quadrants in dark conditions. These patients were considered to PACS cases. PAC occurred if the PACS status was associated with peripheral anterior synechiae and/or intraocular pressure (IOP) of >21 mm Hg. PACG was diagnosed if there were concurrent optic disk and visual field changes characteristic of glaucoma.

All patients had a repeat AS-OCT scan performed a mean of 6 (± standard deviation 3) weeks after treatment with PI. The decision on whether LPI was clinically indicated, was made by the glaucoma specialist (APW) based on the AS-OCT images of all four quadrants, using the same criteria as deciding whether or not to perform a PI. Essentially, those eyes that had residual iridotrabecular contact in 2 or more quadrants in dark conditions post-PI were deemed to still have occludable angles and were selected for LPI.

Exclusion criteria were secondary angle closure (such as angle neovascularization, trauma and intumescent cataract), previous intraocular surgery or poor-quality AS-OCT images that were unsuitable for angle evaluation. As with the original cohort, only the right eye was used for analysis if both eyes were eligible.

**Fig. 1:**
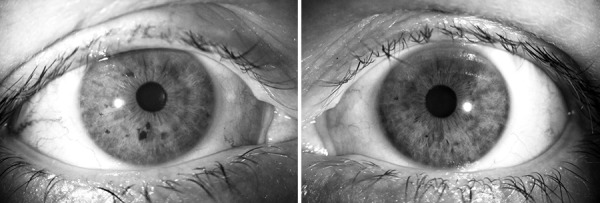
A typical example of a patient treated with unilateral diode laser peripheral iridoplasty. There is mild anisocoria and visible, subtle laser spots in the peripheral iris (left), compared with the fellow untreated eye (right)

LPI was performed using the Oculight SLx diode laser. After pupil constriction with pilocarpine 2%, 30 to 35 shots were applied on to the iris as peripherally as possible over 360°, using a power between 200 and 350 mW, a 2.5 seconds maximum treatment time and a 500 μm spot size. The power and duration (controlled via footpedal) were titrated to be just enough to cause iris contraction but not superficial iris charring (Fig. 1). Typically, a contact lens was not used but occasionally a three-mirror lens was used (avoiding the use of a magnifying lens which would decrease the spot size on the iris and therefore, affect the power exponentially). After LPI, patients were given a 5 day course of topical predni-solone acetate 1.0% to relieve postlaser infammation.

At the follow-up visit, AS-OCT imaging was repeated for each patient, as well as routine clinical examination, which included IOP measurement, gonioscopy and confirmation that the PI has remained patent after LPI. The AS-OCT images before and after LPI were reviewed and analyzed for each patient. Although multiple scans of all four anterior chamber angle quadrants were captured and treatment decisions based on images from all four quadrants, only the horizontal images were analyzed in this study because these provided a clearer view of all the anterior chamber angle structures, in particular the scleral spur and peripheral iris recess, compared to the vertical images.

The nasal and temporal quadrants for all eyes were measured by a single observer a glaucoma fellow (GSA) who was masked to the identity and sequence of the images being evaluated. The observer selected the best quality image with clearly identifable anatomical landmarks from a series of images of the nasal and temporal quadrants, all centered on the pupil, obtained by the ophthalmic imaging technicians. After the locations of the scleral spur and iris recess apex were selected, various anterior chamber drainage angle para meters were calculated utilizing the inbuilt analysis software. These parameters were: trabecular-iris angle (TIA), angle opening distance (AOD), trabecular-iris space area (TISA) and anterior chamber depth. In addition, parameters not included in the inbuilt analyses were measured manually: trabecular-iris contact length (TICL), iris thickness (IT) and maximum iris bow height (MIBH). The observer GSA had previously demonstrated moderate to good intraobserver reproducibility with the intraclass correlation coefficient statistic on all of these AS-OCT parameters in a larger cohort of patients, from which this present cohort was derived from, using the same AS-OCT instrument and analysis software.^13^ Although the AS-OCT images were captured in both light and dark conditions, only results of scans in the dark were used for analysis.

**Figs 2A and B:**
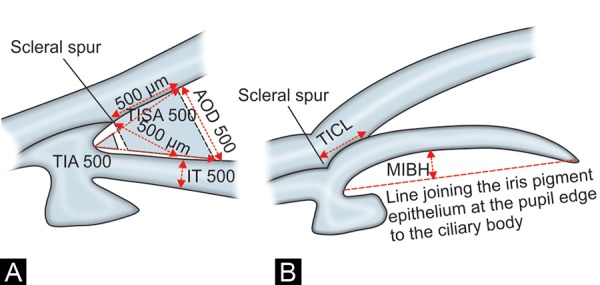
A schematic diagram of anterior segment optical coherence tomography measurement parameters. (A) Trabecular-iris angle (TIA) 500, angle opening distance (AOD) 500 and trabecular-iris space area (TISA) 500. (B) Trabecular-iris contact length

Figures 2A and B show the parameters measured with the AS-OCT. The TIA 500 was the angle between the point of the trabecular meshwork 500 mm from the scleral spur and the point on the anterior iris perpendicularly, with the apex at the iris recess.^19^ The TIA 750 was similar to TIA 500, except that the angle was measured from the point of the trabecular meshwork 750 mm from the scleral spur. AOD 500 and AOD 750 were the perpendicular distances from the trabecular meshwork at 500 and 750 mm, respectively, anterior to the scleral spur to the anterior iris surface.^19^ The TISA 500 was the trapezoidal area bordered anteriorly by the AOD 500, posteriorly by a line from the scleral spur perpendicular to the plane of the inner sclera to the anterior iris, superiorly by the inner corneoscleral wall and inferiorly by the anterior iris surface.^20^ TISA 750 was similar to TISA 500, except that it was bordered anteriorly by the AOD 750. The TICL was the length of contact between the anterior iris surface and the inner corneoscleral wall.^20^ IT 500 was the perpendicular distance from the anterior iris surface at 500 mm from the scleral spur to the posterior iris pigment epithelial surface.^21^ The MIBH was used as a surrogate marker for iris curvature and was the perpendicular distance measured from the posterior iris pigment epithelial surface at its apex (i.e. the point where iris bowing was greatest) to the line joining the iris pigment epithelium at the pupil edge to its insertion at the ciliary body.^22^ The hyper refective curve on the posterior iris surface marked the iris pigment epithelium; its insertion at the ciliary body was the point where the hyperrefective curve terminated within the ciliary body. The secondary outcome measures were IOP and post-LPI complications.

Data were analyzed using Excel software (Microsoft, USA). Basic descriptive statistics was conducted for patient demographics. Comparison of means was performed with the paired t-test for parametric data, while comparison of medians was performed with the Wilcoxon signed rank test for nonparametric data. A p value less than 0.05 was considered to be statistically significant. The study was conducted according to the tenets of the Declaration of Helsinki and had received approval from the Central Regional Ethics Committee of New Zealand.

## RESULTS

All 14 eyes that underwent LPI out of the original PI cohort of 71 eyes met inclusion criteria and did not fulfil any exclusion criteria and were, therefore, included in this study. Mean age at treatment was 55.9 (±10.9) years. A total of 11 patients (79%) were women. Four (29%) had PACG, while the remainder had PAC or PACS. The mean time from LPI to the follow-up scan was 6.3 ± 2.9 weeks. None of the patients had experienced postlaser complications such as persistent pain and/or photophobia, persistent uveitis, or elevated IOP spikes.

**Figs 3A and B:**
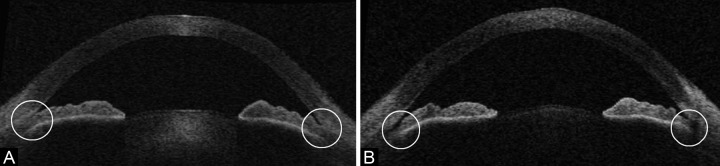
Representative example of a comparison of AS-OCT in a patient pre (A) and post (B) LPI. Note the widening of the anterior chamber drainage angle (circled)

When comparing indicators of angle width (TIA 500, TIA 750, AOD 500, AOD 750 and TISA 500) before and after LPI, there was generally a statistically significant increase in magnitude. The TICL and MIBH showed a statistically significant decrease for both nasal and temporal angles. Iris thickness measurements (IT 500) did not show any meaningful difference post-LPI. These parameters are summarized in Table 1 and an example of the AS-OCT changes after LPI is shown in Figures 3A and B.

The median number of quadrants with persisting iridotrabecular contact on AS-OCT decreased after LPI from 4 to 1 (p < 0.001, Z = –3.236; Wilcoxon signed rank test). There was also a small but statistically significant drop in IOP from 17.1 to 14.8 mm Hg (p = 0.014). The change in the parameters is summarized in Table 2.

**Table 1:** **Table 1:** Changes in anterior chamber and angle parameters post LPI. Various parameters are measured by AS-OCT in dark conditions for both temporal and nasal angles, and mean ± standard deviation for 14 eyes are compared pre and post LPI differences

		*Temporal angle*						*Nasal angle*					
		*Preiridoplasty*		*Postiridoplasty*		*Significance (t-test)*		*Preiridoplasty*		*Postiridoplasty*		*Significance (t-test)*	
TIA 500 (°)		6.07 ± 6.08		13 ± 6.35		0.002		4.077 ± 5.53		13.214 ± 5.82		<0.001	
AOD 500 (mm)		0.055 ± 0.055		0.142 ± 0.073		<0.001		0.033 ± 0.047		0.144 ± 0.057		<0.001	
TISA 500 (mm^2^)		0.038 ± 0.032		0.058 ± 0.034		0.063		0.011 ± 0.016		0.061 ± 0.036		<0.001	
TIA 750 (°)		6.79 ± 4.92		14 ± 6.74		<0.001		6.21 ± 5.85		14.21 ± 5.49		<0.001	
AOD 750 (mm)		0.092 ± 0.068		0.213 ± 0.119		<0.001		0.079 ± 0.081		0.212 ± 0.081		<0.001	
TISA 750 (mm^2^)		0.092 ± 0.068		0.105 ± 0.054		0.408		0.038 ± 0.038		0.110 ± 0.057		<0.001	
TICL (mm)		0.334 ± 0.292		0.082 ± 0.175		0.002		0.586 ± 0.29		0.176 ± 0.174		<0.001	
IT 500 (mm)		0.442 ± 0.183		0.421 ± 0.067		0.510		0.432 ± 0.053		0.410 ± 0.103		0.423	
MIBH (mm)		0.158 ± 0.090		0.085 ± 0.113		0.017		0.158 ± 0.092		0.063 ± 0.079		<0.001	

**Table 2:** **Table 2:** Changes in intraocular pressure, anterior chamber depth and number of quadrants with persisting iridotrabecular contact on AS-OCT before and after laser peripheral iridoplasty

		*Preiridoplasty*		*Postiridoplasty*		*Significance*	
Mean intraocular pressure (IOP)		17.11 ± 4.01		14.78 ± 4.55		0.014 (paired t-test)	
Anterior chamber depth (ACD)		2.12 ± 0.43		2.18 ± 0.34		0.34 (paired t-test)	
Median number of quadrants closed		4		1		<0.001 (Wilcoxon signed rank test)	

## DISCUSSION

This study represents to the best of our knowledge, the only study to demonstrate with AS-OCT, the quantitative changes in the anterior chamber angle following LPI. Our results demonstrate that in the short-term, in this cohort of patients with residual iridotrabecular contact after PI, additional LPI brings about significant widening of the anterior chamber angle as measured by AS-OCT. This was demonstrated by the significant increase in TIA and AOD for the temporal and nasal angles, measured at both 500 and 750 μm from the scleral spur. Interestingly, although the TISA 500 and 750 increased for both nasal and temporal angles, the change observed did not reach statistical significance for the temporal angle. It is possible that this difference did not reach statistical significance because of our small study sample size. TICL was significantly reduced and IT was not affected. The failure to detect a difference in iris thickness following LPI may be due to images and measurements being taken at cross sections which did not include LPI spots, where maximal iris stromal thinning would be expected to occur. LPI also appeared to bring about a fattening of the iris contour, with a significant decrease in MIBH. This change to peripheral iris contour provides confirmation of the theoretical mechanism of LPI in causing contraction of the peripheral iris away from the trabecular meshwork.

It is well-established that PI brings about significant changes in various parameters of the anterior chamber angle and numerous studies have previously demonstrated this using AS-OCT as the imaging modality.^13,15,16^ However, changes in the anterior chamber angle after LPI have not been investigated using AS-OCT. Lee et al are the only group to assess imaging characteristics of the anterior chamber in a similar clinical scenario using Scheimpfug imaging but not with AS-OCT.^23^ The authors compared two groups: one that had PI alone and a second that received LPI in combination with PI. The authors used a surrogate measure of midperipheral anterior chamber depth to assess the angle and found a greater increase in angle widening in those having combined LPI and PI compared to those having PI alone.

AS-OCT has emerged as a useful tool in the assessment of the anterior chamber angle and indeed has been shown to have a higher degree of sensitivity in the detection of angle closure when compared to gonioscopy.^24^ It has numerous advantages over other means of quantitatively assessing the angle, namely UBM and Scheimpfug photography. While Scheimpfug photography measures certain anterior segment characteristics well, it does not image the drainage angle fully due to the inability of the camera light to penetrate the corneoscleral limbus. UBM has one major advantage over AS-OCT in that it is superior in assessing features of the ciliary body, which may have particular importance in this cohort of patients, in whom angle closure remains despite resolution of pupil block. AS-OCT, however, has a number of advantages. It is less operator-dependent and hence easier to use, has a higher degree of spatial resolution, and is a noncontact test. Its noncontact nature is beneficial for both the patient and clinician; it is more comfortable and potentially safer for the patient, while from the clinician's perspective, there is less likelihood of external pressure affecting anterior segment anatomy. AS-OCT has been shown to have good reproducibility and repeatability, as well as low interobserver variability,^25-27^ and has been demons trated to be similar to UBM when measuring drainage angle parameters quantitatively.^20^ Our observer, GSA, had previously demonstrated moderate to good intraobserver with the AS-OCT instrument and measurement parameters used in this study.^13^

The role of LPI for the treatment of angle closure in the nonacute setting is unclear, but there remains a consensus that it is a useful adjunct in the setting of persistent occlu dable angles after PI.^10^ Despite this consensus, to date the evidence of its overall efficacy in the clinical setting is lacking.^9^ Only one randomized controlled trial has been published comparing PI alone to PI in combination with LPI, and this study showed no superiority to use of LPI as an adjunct to PI for IOP control, number of medications or requirement for surgery.^28^ This was in contrast to our results where there was a small but statistically significant decrease in IOP after LPI. This difference in IOP outcome may be due to a more severe spectrum of angle closure in the above trial population with participants required to have fulfilled criteria for PAC or PACG. Our study included patients with PACS and was not specifically designed to assess IOP outcomes following LPI.

This study has a number of limitations, including the retrospective study design and relatively small number of patients. It was not possible to determine whether the exact, same cross section of the anterior chamber angle was imaged before and after LPI, although it was endeavored to center the AS-OCT images on the pupil in the central horizontal meridian for all images to achieve maximum consistency. Bias could have been introduced during measurements of the angle parameters as the measuring observer (GSA) was not masked as to whether the images were pre- or post-LPI, but this is mitigated by his being masked to the identity and sequence of the images being evaluated.

In addition, the image resolution of the time domain AS-OCT platform used in this study may not have been sufficient to identify the individual iridoplasty spots. It is plausible that if the scan cross section was through one of these spots, there may be local discrepancies in angle configuration in these areas which may not be present globally throughout the entire circumference of the drainage angle. However, the theoretical mechanism of LPI is for some degree of circumferential contraction of the peripheral iris to occur due to 360° spot application. Therefore, a follow-up AS-OCT image may not have to be centered directly across a LPI spot for a difference in angle configuration to be detected. This study only utilized scans from the nasal and temporal quadrants due to the relative difficulty identifying anatomical landmarks consistently in superior and inferior quadrants; a methodological issue that has previously been addressed in a similar fashion by other groups.^15,29,30^ The spectral domain OCT platforms which have been introduced more recently may allow for quicker image acquisition, higher resolution, and image averaging as well as more accurate referencing of the location of each cross sectional scan. A further limitation of AS-OCT is its inability to directly evaluate the ciliary body, such as for rotation, which would have been of particular interest in this cohort of patients. This study does not report gonioscopic findings in the study cohort as the aim is to quantify changes in angle configuration using AS-OCT.

## CONCLUSION

This study describes systematically for the first time the changes observed by AS-OCT in the anterior chamber angle configuration induced by LPI in a cohort of patients with residual angle closure after PI. Our data confirm an additional anterior chamber angle widening effect and a small IOP lowering effect with LPI, at least in the short term. Long-term follow-up of this cohort will be useful to longitudinally assess the longevity of the structural and IOP changes, following LPI and its relevance in terms of glaucoma progression.
